# A DIGITAL ELDER ABUSE INTERVENTION FOR THE EMERGENCY DEPARTMENT

**DOI:** 10.1093/geroni/igac059.1669

**Published:** 2022-12-20

**Authors:** Fuad Abujarad, Esther Choo, James Dziura, Chelsea Edwards, Michael Pantalon, Karen Jubanyik, Gail D'Onofrio, Thomas Gill

**Affiliations:** Yale University, Orange, Connecticut, United States; Oregon Health & Science University, Portland, Oregon, United States; Yale University, New Haven, Connecticut, United States; Yale University, New Haven, Connecticut, United States; Yale University, New Haven, Connecticut, United States; Yale University, New Haven, Connecticut, United States; Yale University, New Haven, Connecticut, United States; Yale School of Medicine, New Haven, Connecticut, United States

## Abstract

Elder abuse continues to grow as a national issue with significant gaps in standards for identifying victims. The emergency department provides an opportune setting for affected older adults to report abuse privately, but traditional screening methods may miss certain types of elder abuse when there are no visible signs of abuse. Community resources and reporting methods may not be known or can be stigmatized, therefore creating a challenging scenario for victims of abuse to navigate without fear. The VOICES Elder Abuse Intervention (EAI) is a unique self-administrated tablet-based tool with an automated digital coach that combines major components of screening, educational content, and brief psychoeducational interviewing to empower the older adults and encourage self-identification and self-reporting of abuse. The digital format of the tool allows this EAI to be integrated within an existing elder abuse workflow protocol in the emergency department, rather than replacing it entirely. We will discuss our completed study that included (N=1,002) participants over the age of 60, which suggests that patients find the EAI user-friendly and easy to use. For example, 93% of participants agreed that VOICES was appropriate for learning about abuse, and 94% agreed that using VOICES was very easy to use. Informed by these findings, we plan to adapt and evaluate VOICES EAI for use in other older adult populations and healthcare settings.

